# Application of homogenization methods for Ireland's monthly precipitation records: Comparison of break detection results

**DOI:** 10.1002/joc.6575

**Published:** 2020-04-16

**Authors:** John Coll, Peter Domonkos, José Guijarro, Mary Curley, Elke Rustemeier, Enric Aguilar, Séamus Walsh, John Sweeney

**Affiliations:** ^1^ Irish Climate Analysis and Research Units, Department of Geography Maynooth University Maynooth Ireland; ^2^ Centre for Climate Change (C3) Universitat Rovira i Virgili Tortosa Spain; ^3^ Agencia Estatal de Meteorologia Delegación Territorial en Illes Balears Palma, Mallorca Spain; ^4^ Climatology and Observations Division Met Éireann Dublin Ireland; ^5^ Department of Hydrometeorology Deutscher Wetterdienst Offenbach Germany; ^6^ Centre for Climate Change (C3) Universitat Rovira i Virgili Tarragona Spain

**Keywords:** ACMANT, AHOPS, break detection, CLIMATOL, HOMER, homogenization, Ireland, precipitation

## Abstract

Time series homogenization for 299 of the available precipitation records for the island of Ireland (IENet) was performed. Four modern relative homogenization methods, that is, HOMER, ACMANT, CLIMATOL and AHOPS were applied to this network of station series where contiguous intact monthly records range from 30 to 70 years within the base period 1941–2010. Break detection results are compared between homogenization methods, and coincidences with available documentary information (metadata) were analysed. The lowest (highest) number of breaks were detected with HOMER (ACMANT). Large differences of break frequency were found, namely ACMANT and AHOPS detected 8 times as many breaks than HOMER, while the break frequency with CLIMATOL was intermediate. Also, the ratio of series classified to be homogeneous varies widely between the methods. It is 85% with HOMER, 60% with CLIMATOL, 31% with AHOPS, while only 22% with ACMANT. In a further experiment, all the available time series for Ireland and Northern Ireland, (910 series) were used with ACMANT and CLIMATOL to explore the stability of break frequency for the same 299 series examined in the base experiment. While overall break frequency slightly increased (by 6–13%), the break positions notably changed for individual time series. The number of breaks changed for 59% (23%) of the series with ACMANT (CLIMATOL). For the breaks detected coincidentally by at least three methods including ACMANT and CLIMATOL in the base experiment, the second experiment confirmed the break positions in 86–87% of the breaks. The consequences of these results in relation to the reliability of statistical homogenization are discussed. Sometimes markedly different step functions provide comparable good approaches. However, the accuracy of homogenized time series cannot be related directly to the instability of break detection results.

## Graphical abstract

We found 116 concordant breaks indicated with at least three homogenization methods including metadata analysis. This together with some other aspects of the results suggests that the break frequency is higher than in several other European precipitation datasets. However, spatial correlations are generally higher in IENet than in many other precipitation datasets. Additionally, the ratio of series found to be homogeneous and the frequency of detected breaks are strongly method dependent. In the figure, ratios of homogeneous series for the four programmes. Homogeneous series are denoted in blue; series with one detected break in yellow and series with multiple breaks detected in red.

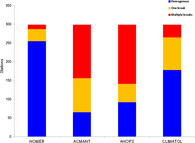

## 1 INTRODUCTION

Climate change studies based only on raw long‐term data are potentially flawed due to the breaks introduced from non‐climatic sources, therefore quality controlled and homogenized climate data are needed for improved climate‐related decision making. Fundamentally the quality of long‐term climate analysis depends on the homogeneity of the underlying time series (Vertačnik *et al*., 2015); and this need for homogeneity also reflects a growing demand for climate services more generally (e.g., Buontempo *et al*., 2014; Vaughan and Dessai, 2014), sometimes expressed as “actionable knowledge” (Asrar *et al*., 2013; Kirchoff *et al*., 2013) for use across a range of decision‐making environments.

A homogeneous climate time series is defined as one where variability is only caused by changes in weather or climate (Freitas *et al*., 2013). Most decade to century‐scale time series of atmospheric data have been adversely impacted by inhomogeneities caused by, for example, changes in instrumentation or observer practices, station moves, or changes in the local environment (e.g., urbanization). Some of these factors can cause abrupt shifts; others gradual changes over time, which can hamper identification of genuine climatic variations or lead to erroneous interpretations (Peterson *et al*., 1998). Since these shifts are often of the same or greater magnitude as the climate signal (Auer *et al*., 2007; Menne *et al*., 2009), a direct analysis of the original data series can lead to incorrect conclusions about the evolution of climate. Seasonal cycles of precipitation in Ireland are projected to become more pronounced as the climate changes (e.g., Nolan, 2015), and regional extremes in summer dry spells and winter heavy rainfall events have been recorded in recent years (Nolan *et al*., 2013). Therefore, to analyse and monitor the evolution of precipitation patterns across Ireland, quality controlled and homogeneous climate series are needed.

Homogeneity tests can be broadly divided into “absolute” and “relative” methods. The former are applied directly to individual candidate stations to identify statistically significant shifts in the section means (referred to as breaks or change points), while relative methods entail comparison of correlated neighbouring stations with a candidate station to test for homogeneity. Thus, relative homogenization algorithms use the difference time series of a candidate station with neighbouring stations to identify such breaks. Reference series, which have ideally experienced all of the broad climatic influences of the candidate but no artificial biases, are commonly used to detect inhomogeneity in relative methods (World Meteorological Organisation (WMO), 2011), as well as to assess the quality of the homogenization process (Kuglitsch *et al*., 2009). Reference series themselves do not need to be homogeneous in modern homogenization methods (Szentimrey, 1999; Zhang *et al*., 2001; Caussinus and Mestre, 2004), but must encompass the same climatic signal as the candidate (Della‐Marta and Wanner, 2006). Relative homogenization is more robust than absolute methods provided station records are sufficiently correlated (Venema *et al*., 2012) and that by definition absolute homogeneity is incompatible with climate change. However, relative approaches can be confounded by lack of long records at neighbouring stations for comparison, and by simultaneous changes in measuring techniques across a network (Peterson *et al*., 1998; Wijngaard *et al*., 2003).

Homogeneity approaches benefit from reliable documentary information of a station's operational history (metadata) to account for breaks and potential outliers. Metadata can provide information such as location of station instruments, when and how observations were recorded, notes on instrument changes and malfunctions or any environmental changes such as vegetation encroachment at the site (Aguilar *et al*., 2003). This information is often useful in interpreting statistical homogeneity tests and for informing the nature and magnitude of adjustments that might be applied to data.

New techniques are emerging for the detection and adjustment of inhomogeneity in climate series (Domonkos, 2011a; 2011b; Cao and Yan, 2012; Toreti *et al*., 2012; Freitas *et al*., 2013; Mestre *et al*., 2013) and the correction of multiple change points using reference series. Modern multiple breakpoint methods search for the optimum segmentation characterized by minimum internal variance within the segments and maximum external variance between the segment means (Caussinus and Mestre, 2004; Lindau and Venema, 2016). A comprehensive assessment of homogenization techniques for climate series was included in the scientific programme of the COST Action HOME ES 0601 *Advances in Homogenization Methods of Climate series: An integrated approach*. The HOME objective was to test the existing statistical homogenization techniques and develop more efficient methods for homogenizing climate datasets. PRODIGE (Caussinus and Mestre, 2004) was one of the best performing methods at that time. Both its break detection and correction methods gave outstanding results (Domonkos *et al*., 2011; Domonkos, 2013), and it was also one of the most accurate methods in the blind test experiments (Venema *et al*., 2012). Relying on the HOME blind test experiments, five homogenization methods were recommended to use: PRODIGE, ACMANT (Domonkos, 2011a), MASH (Szentimrey, 1999), USHCN (Menne and Williams, 2009) and the graphical method of Craddock test (Craddock, 1979).

After the HOME blind tests, the methodological developments were continued. In this study, four relatively new methods, namely HOMER (HOME, 2013; Mestre *et al*., 2013), ACMANT (Domonkos, 2015; Domonkos and Coll, 2017a), CLIMATOL (Guijarro, 2018) and AHOPS (Rustemeier *et al*., 2017) will be applied. Note that although ACMANT and CLIMATOL were tested during HOME, their new versions markedly differ from the ones tested at that time. Three of the four methods, that is, HOMER, ACMANT and AHOPS are developed from PRODIGE, keeping the principal detection and correction methods of PRODIGE, but adding also new routines and new features to them.

The aim of the study is twofold: (a) We will examine the operation of the four modern homogenization methods on a rather large precipitation dataset complicated by varied lengths of observed time series and large data gaps. (b) We will examine the degree of instability in break detection results according to station density and homogenization methods, and analyse the relation between this instability and the practical applicability of the selected methods. All of the four methods will be examined for a medium‐sized station network and two of the four will also be examined with a denser station network. Note that although the final purpose of homogenization is not the break detection but the removal of non‐climatic biases from the observed data, the break detection is an important step (Venema *et al*., 2012; Lindau and Venema, 2013, 2016), and break detection errors may seriously affect the final accuracy of homogenization products (Lindau and Venema, 2018a). Note also that in homogenization tasks including the exhaustive analysis of station history, the skill of break detection may have a large and direct impact on the accuracy of the homogenization results.

## 2 DATA AND METHODS

### 2.1 Study area

**FIGURE 1:**
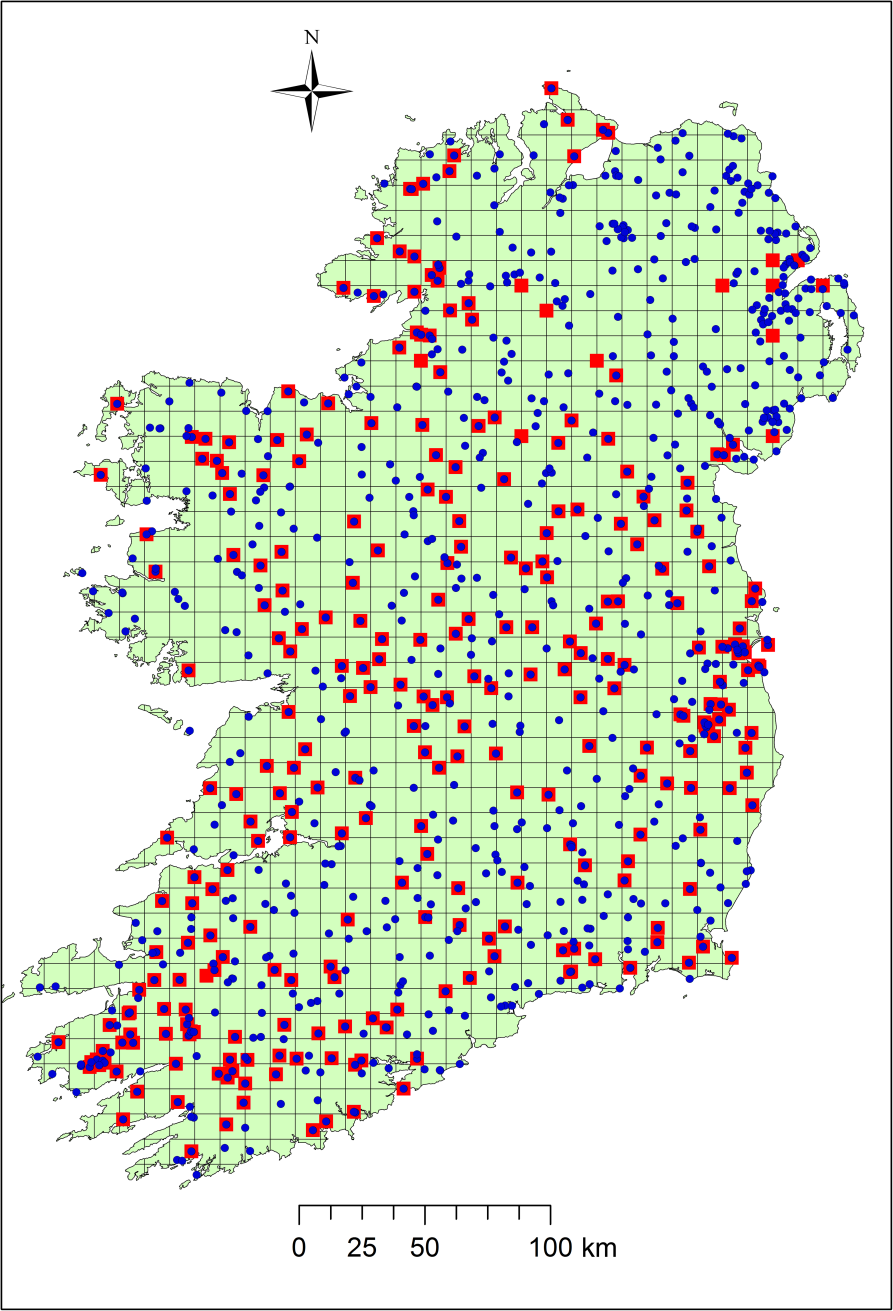
Annotated map of the island of Ireland showing the selected Met Éireann and Met Office, United Kingdom monthly station locations for the network of (a) 299 stations (sub‐IENet) denoted by red squares; (b) 910 stations (whole‐IENet) denoted by blue circles. Station marks are overlain on a regular 10 × 10 km grid to give an indication of density

The study area is the whole island of Ireland, that covers ~84,421 km^2^ on the Atlantic margin of northwest Europe, between ~51° and 56°N (Figure 1). Elevations reach up to 1,038 m above sea level (a.s.l.; Carrauntoohil, Co. Kerry). Much of the island is lowland, partly surrounded by mountains, with a characteristic temperate oceanic climate. Associated with Ireland's maritime location and the prevailing wind direction, the bulk of precipitation comes primarily from the Atlantic southwest and to a lesser extent from the northwest, whereas cold and dry weather comes from the east and continental Europe. On average, annual precipitation ranges from 750 to 1,000 mm in the drier eastern half of the country and >3,000 mm·year^−1^ in parts of the western mountains (Rohan, 1986). Although some snowy days occur in winter and early spring, the majority of precipitation is as rainfall year round.

### 2.2 Source data

Rainfall has been measured in Ireland since the early nineteenth century with a peak of over 800 rainfall stations in the late 1950s. Currently, rainfall is recorded at synoptic and climatological weather stations; in addition, there is a wide network of voluntary rainfall observers (Walsh, 2012). The total dataset of IENet consists of 703 time series of Ireland and further 207 time series of Northern Ireland (NI). NI data were provided to us by the UK Met Office. Station elevations are within the range of 5–701 m a.s.l. with a mean elevation of 78 m.

**FIGURE A1:**
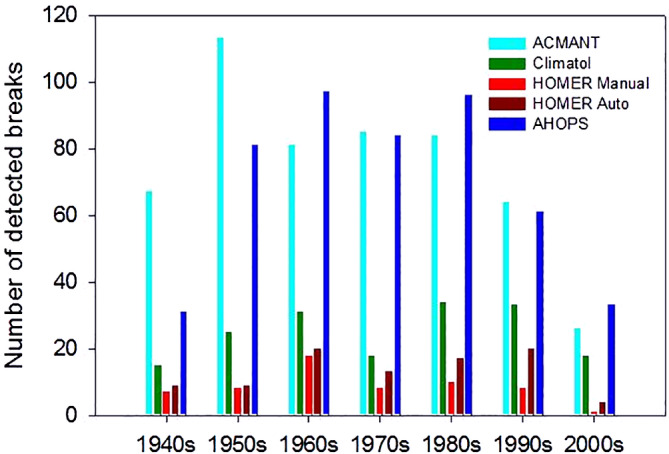
Break detection count summary for the four methods for sub‐IENet by decades (the 1940s start in 1941). HOMER manual and automatic (Auto) break counts included for comparison

Following an audit and quality control on the data, an exploratory statistical analysis of the data was undertaken to characterize the properties of the series, as well as to identify missing values and outliers. Figure A1 provides a HOMER‐derived summary of the extent of the intact months and missing records across all the stations in the network. Issues with missing data in climate time series can be tackled with spatial interpolation using data from nearby stations (WMO, 2011). However, in the homogenization here we use only the raw data with missing values retained, since gap filling before a homogenization procedure can lead to deterioration of the homogenization results (Auer *et al*., 2005; Domonkos and Coll, 2019).

Based on experience with previous networks and HOMER's and AHOPS's lower tolerance of missing values than some of the other methods, short or fragmented series were excluded. As a result of this restriction, a subset of 299 station series have remained, 287 stations for Ireland and 12 NI stations (Figure 1). For each series of this subset (referred to as sub‐IENet hereafter), the contiguous intact monthly records ranges from 31 to 70 years for the 1941–2010 period. Only sub‐IENet is used in the study except when the text indicates the use of the entire IENet. Approximately two thirds of the selected stations have metadata support. Despite the exclusions, with a mean density of ~0.003 stations per km^2^ sub‐IENet is more dense than for HOMER network experiments reported elsewhere (Vertačnik *et al*., 2015; Osadchyi *et al*., 2018 for temperature; Prohom *et al*., 2016; Pérez‐Zanón *et al*., 2017 for precipitation). While Gubler *et al*. (2017) used HOMER to homogenize precipitation and temperature series, their denser Swiss precipitation network was still sparser than sub‐IENet.

### 2.3 Break detection in relative homogenization methods

Break detection is an important step of time series homogenization. The base idea of break detection with relative homogenization is that via the generation of differences or ratios of a candidate series and reference series of nearby stations, the regionally common climate signal will be removed from the resultant relative time series. The general model of relative time series (*T*), includes terms for both station effect (*S*) and Gaussian noise (*ε*). For a time series of length *n* containing *K* break points, the relation is shown by Equation (1). If the seasonal cycle is removed before the generation of *T*, cyclical terms do not complicate the equation,

(1) $$T_{i}=S\left(k\right)+\varepsilon_{i};i=1,2,\ldots n;k=1,2,\ldots K+1.$$

As we examine monthly precipitation series, the time unit is a month, or in certain steps of a break detection procedure it can be year. If a station series is homogeneous, then its station effect is constant for the entire series. If it is inhomogeneous, as almost all inhomogeneities are sudden shifts of the means, then the station effect will be a step function of *K* steps including *K* + 1 sections with constant values. Even when some inhomogeneities are not sudden shifts (but, e.g., gradual changes), this step function model gives good results (Domonkos, 2011b), and is used in the methods involved in our study. Station effect may hold seasonal differences depending on, for example, the proportion of winter precipitation received as snow as opposed to liquid precipitation. For regions without snow or with little snow in winter (as in our case), the exclusion of this seasonality from the model is widely recommended for the homogenization of precipitation totals (Auer *et al*., 2005; Moisselin and Canellas, 2005; Domonkos, 2015) for the typically too low signal to noise ratio (SNR).

The principal task for break detection is to make a distinction between the station effect and background noise. The noise term of Equation (1) is the composition of weather and non‐systematic observation errors. For monthly or annual time series the noise is approximately white noise (Hannart *et al*., 2014; Lindau and Venema, 2016). However, since the climate is spatially coherent, the true noise is temporally not fully independent either on long time scales. The true content of *ε* includes the spatial difference between the climate of the candidate series and that of the reference series. For this climate effect, *ε* tends to have persistence and is often modelled with a first order autoregressive process (Lund *et al*., 2007; Wen *et al*., 2011). However, in a separate Europe‐wide application of AHOPS, autocorrelation for monthly precipitation was found to be low and not significant when the annual cycle is removed (Rustemeier *et al*., 2017).

The break detection methods of HOMER, AHOPS and CLIMATOL are based on white noise background noise, while ACMANT includes empirical parameterisation (Domonkos and Coll, 2017a) adapted from the homogenization of temperature test datasets of Venema *et al*. (2012) and Willett *et al*. (2014), hence any presumption about the content of *ε* is avoided there.

### 2.4 Homogenization methods

Break detection is performed with four homogenization methods. Three of the methods, that is, HOMER, ACMANT and AHOPS include the optimal step function fitting (optimal segmentation; Caussinus and Mestre, 2004) with dynamic programming (Hawkins, 1972; 2001) for break detection and the network wide minimization of residual variance for correcting inhomogeneities (ANOVA correction; Caussinus and Mestre, 2004; Mamara *et al*., 2014; Domonkos, 2015). These techniques are known to be some of the most effective statistical tools in the homogenization of climatic time series with multiple breaks (Caussinus and Mestre, 2004; Domonkos *et al*., 2011; Domonkos, 2013). All of the three methods of HOMER, ACMANT and AHOPS use some derivatives of the Caussinus–Lyazrhi criterion (Caussinus and Lyazrhi, 1997) for calculating the number of homogeneous segments. This criterion is based on information theory and the penal term included in it prevents the inclusion of insignificant breaks. In spite of a lot of similarities, some important differences exist between these methods. The most important differences are that while ACMANT and AHOPS use composite reference series (Peterson and Easterling, 1994; Alexandersson and Moberg, 1997) for the time series comparison, HOMER applies pairwise comparison (Menne *et al*., 2009; Dunne *et al*., 2014) allowing a detailed analysis of individual breaks and user intervention when it is beneficial.

CLIMATOL uses composite reference series and detects breaks one‐by‐one with the Standard Normal Homogenization Test (SNHT; Alexandersson and Moberg, 1997). Series are split at the detected break‐points, and then the detection process continues with the search for possible further breaks in the subseries. The core idea of this algorithm is well established, and the hierarchic break detection algorithms applied are often considered theoretically inferior in comparison to modern multiple break detection algorithms (Mestre *et al*., 2013; Domonkos, 2017; Szentimrey, 2017). However, efficiency tests show that CLIMATOL is competitive with multiple break methods in the accuracy of the homogenization product delivered (Killick, 2016; Guijarro *et al*., 2017).

HOMER is better suited to small to medium sized networks with the use of metadata, while ACMANT, AHOPS and CLIMATOL are excellent tools for the automatic homogenization of large and dense networks or the homogenization of any network when metadata is not available, although CLIMATOL also has the facility to incorporate metadata information. In this study, only the HOMER method is applied together with metadata use.

#### 2.4.1 HOMER

The HOMER (HOMogenization softwarE in R) package was a key deliverable of the COST action HOME and represents a synthesis of homogenization approaches (Mestre *et al*., 2013), including some homogenization routines of PRODIGE (Caussinus and Mestre, 2004), ACMANT (Domonkos, 2011a) and the network wide joint segmentation method of Picard *et al*. (2011), as well as some common quality control and visualization routines of the CLIMATOL homogenization method (Guijarro, 2018). HOMER is an interactive, semi‐automatic method for homogenization where the user can take advantage of available metadata in the detection and correction of time series (Vertačnik *et al*., 2015).

In the pairwise comparison, series are compared with all other series from the same climate region to produce series of differences between the candidate and others in a defined network. Difference series are then tested for change points (Mamara *et al*., 2014). Once detected breaks have been checked against metadata, non‐homogeneous series are corrected using the ANOVA model.

Creation of a reference network for a given candidate station is a key step in the homogenization process. The network can be defined based on geographic proximity or station correlation. To ensure that candidate stations have sufficient reference stations for each year of the series, it is necessary to set the minimum number of reference stations (Vertačnik *et al*., 2015). Previous work applying HOMER to monthly precipitation data in Ireland revealed that geographical or correlation distance selections in HOMER yield overlapping neighbour series which are largely statistically and spatially coherent (Coll *et al*., 2014; Noone *et al*., 2015). Such a relationship is specific for geographical zones of maritime climate, where the range of variation in precipitation totals is lower than for a continental climate (Coll *et al*., 2015).

For the analysis reported here we chose to use the geographical distance selection, as in our experience high numbers of missing values may be creating spurious correlations in some of the sub‐networks identified by HOMER. For each candidate station, the closest 15 stations were selected. These sub‐networks facilitated the homogenization and completion of all series to the common period of 1941–2010 while avoiding a known limitation of the software to correct when there are many blocks of missing contiguous data distributed across candidate and/or reference series (Coll *et al*., 2015).

We adopted a three‐stage application of HOMER to allow greater scrutiny of detected inhomogeneities before the corrections were applied. First, basic quality control and network analysis were performed. Outliers were identified using both HOMER and visual inspection and by defining minimum and maximum monthly outliers as values exceeding ±1.96 standard deviations from the respective series mean (Coll *et al*., 2018). Outliers were checked against a number of the nearest reference stations as well as metadata and any likely cases were removed. Second, HOMER was run to identify breaks within each time series. The break detection comprised five iteration steps of Pairwise Detection (PW) → Joint Detection (JD) → PW → JD → PW. Detected breaks were not corrected automatically; all were checked for consistency with the relevant reference stations and by scrutiny of metadata. Third, following confirmation of breaks with available metadata HOMER was used to correct series for inhomogeneity and infill data gaps. Following the recommendations of HOME (2013) we applied multiplicative corrections. The correction factors were constant within calendar years for the reasons discussed in section 2.3.

#### 2.4.2 ACMANT

ACMANT is a fully automatic homogenization method, and one where an automatic control of monthly outlier values is provided based on the spatial comparison of simultaneous data. Infilling of missing data is a part of the ACMANT procedure; however, there is also an option for the user to choose if the homogenized output with the completed data is required, or whether the existing gaps associated with the raw data being retained are preferred. In this study, ACMANTv3 (Domonkos and Coll, 2017a) is applied. From this third generation of ACMANT, the method includes an ensemble pre‐homogenization whereby the minimal adjustment term is always retained among the adjustment terms derived from individual ensemble members. A further novelty of ACMANTv3 is that the weighting of reference composites includes ordinary kriging (Szentimrey, 2008; Domonkos and Coll, 2017a).

Automatic networking (AN) has been developed (Domonkos and Coll, 2019) as a preparatory operation for homogenizing datasets of larger than 40 series with the ACMANT method. In AN a specific network is constructed for each candidate series which provides optimal spatial comparison with the candidate series always in the centre of the network (Domonkos and Coll, 2017b). The inclusion of AN increases the computational time demand as the number of networks equal the number of time series in the dataset, but the overall computational time demand of ACMANT still remains below tolerable limits. Note that AN can also theoretically be used with any other homogenization method when the computational time demand remains feasible for a large number of networks of 30 to 60 time series. Most recently, ACMANTv4 is also available for interested users (Domonkos, 2019) together with the full description of its scientific content (Domonkos, 2020).

#### 2.4.3 AHOPS

The Automatic HOMogenization of Precipitation Series (AHOPS; Rustemeier *et al*., 2017) is also developed from PRODIGE, and similarly to ACMANT, break‐points are detected via automated comparisons with a selected group of reference series. As Pearson correlation has a limited application for variables with strongly asymmetric distributions, AHOPS uses partial rank correlations for network formation, and applies WARD's method for finding the clusters of minimum variance (Wilks, 2006). Once these clusters have been established, precipitation amounts are converted to a semi‐Gaussian additive variable by logarithmic transformation, and Spearman correlations of the increment series are considered during the selection of the reference series within clusters, which is repeated in each iteration step. For correction of the monthly values the Box‐Cox transformation is applied to convert the precipitation values into an additive variable. The break detection and bias correction methods are the same as for those in HOMER and ACMANT. AHOPS' detection works on an annual scale to reduce the white noise and the temporal resolution of the break positions is annual. Through an ensemble approach AHOPS also displays the reliability of the detected breaks and the homogenized series.

AHOPS is an iterative method. The break detection and monthly bias corrections are repeatedly applied, firstly with raw reference series, and in the later steps of the procedure with pre‐homogenized reference series. The iteration terminates when the break positions become stable. An additional two sample Wilcoxon significance test to filter only the significant break‐points was disabled to see the actual AHOPS performance.

#### 2.4.4 CLIMATOL

CLIMATOL is a set of routines for processing climatological data in R. The method is based on comparing each test series with a reference series through interpolation of ratios (the option used in this study), differences or standardized values of the closest surrounding data at each time step (Guijarro, 2018). This approach has the advantage of being both robust and simple. The method also allows data from nearby stations to be used in cases where there is no common period of observation (Guijarro, 2018) and applies the use of the Reduced Major Axis model (Clark, 1980), a kind of Orthogonal Regression. However, this method requires the means of the series for the entire study period to be known, which is clearly problematic where there is missing data. Therefore, and to counter this, the first normalization of the series is done with means computed only with the available data in each series. Then missing data are estimated from them, new means are calculated, and this procedure is repeated until the means converge. Following these procedures the shift detection is addressed by applying the SNHT test to the series of anomalies (differences between the observed and estimated data) and splitting the series at the detected break‐points. This process is also iterative, in order to remove the major inhomogeneities in the first place, and to refine the detection until no SNHT value over a prescribed threshold is found. At this point all homogeneous sub‐series are completed in their entirety, that is, all missing data are infilled by means of the weighted mean of the closest four sets of normalized data. This approach was chosen in order to be able to use almost all the information of a network, including short series.

### 2.5 Comparison of the break detection results

First the series comprising sub‐IENet are used to homogenize all the series in that network, while in the second part of the analysis, again the sub‐IENet series are homogenized, but the other series of the whole‐IENet are also used as reference series. This secondary analysis for the denser network is performed only with ACMANT and CLIMATOL, since HOMER and AHOPS were unable to cope with the short and fragmented series associated with the wider IENet. The objective of using the two different parent datasets is to examine the effect of network density on the results.

The following statistical characteristics are examined for method comparisons: frequency of detected breaks, the ratio of time series found to be homogeneous, and the magnitude of detected breaks. Break magnitudes are only provided for HOMER and ACMANT, and this is pragmatic as the other methods do not readily yield the data on the break magnitudes.

## 3 RESULTS

### 3.1 Homogenization with the use of sub‐IENet (299 stations)

#### 3.1.1 Break frequency

**FIGURE 2:**
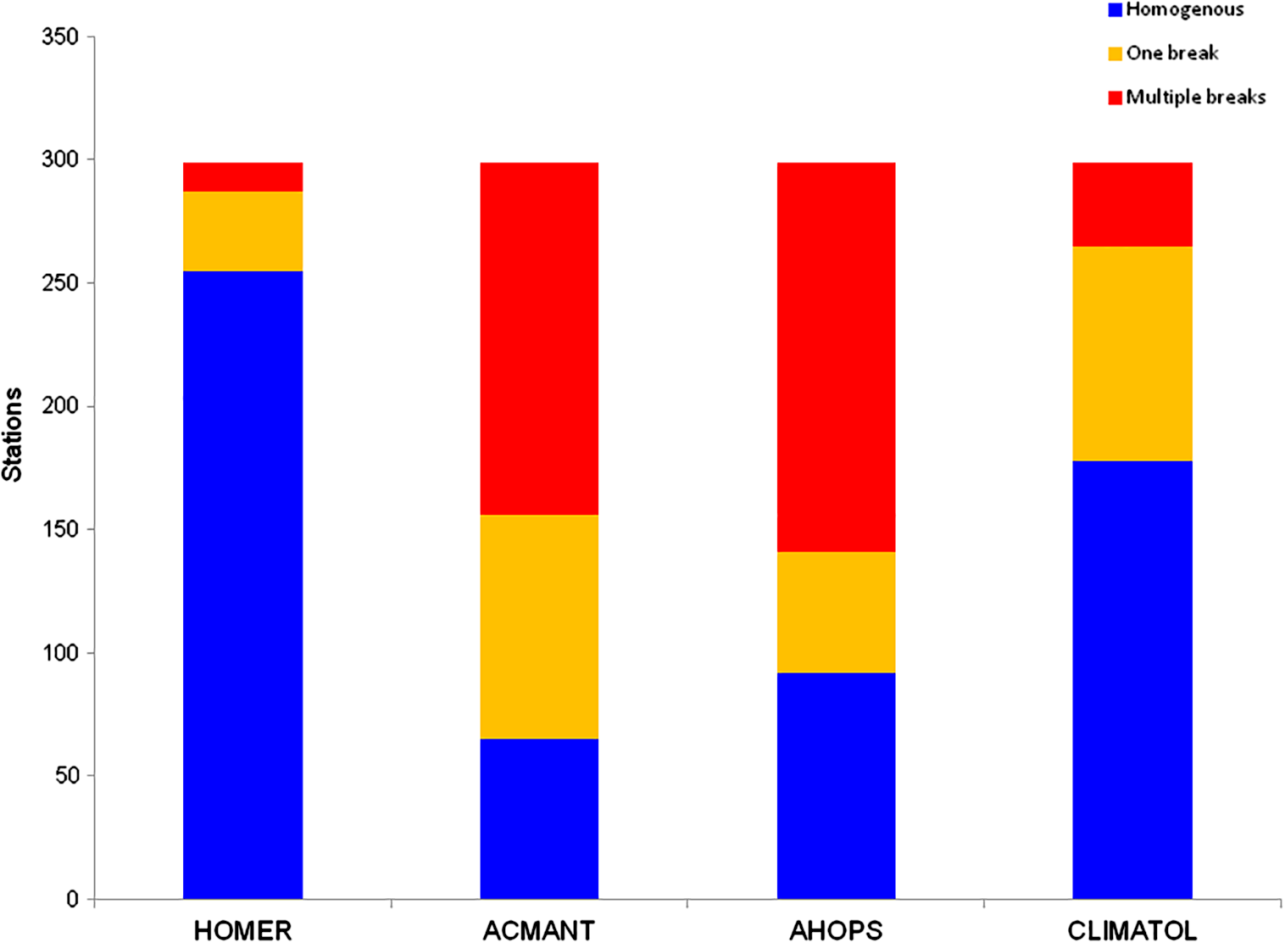
Ratios of homogeneous series for the four programmes. Homogeneous series are denoted in blue; series with one detected break in yellow and series with multiple breaks detected in red

Figure 2 shows the ratio of homogeneous series, series with one break and series with multiple breaks, according to the four homogenization methods applied. There is considerable diversity in the results. With HOMER, 255 series (85%) were found to be homogeneous, while multiple breaks were detected in only 12 records (4%). By contrast, with ACMANT and AHOPS the ratio of homogeneous series is only 22 and 31%, respectively, and multiple breaks were found in approximately half of the series. CLIMATOL has intermediate results with its 178 homogeneous series (60%) and 34 series with multiple breaks (11%). Note, however, that HOMER was used interactively, and 32 potential breaks were removed based on operator interpretation of the metadata and reference series evidence. This removal contributed to the relatively low number of detected breaks with HOMER, although even with the break counts detected in automatic mode left in, HOMER still detected less breaks than the other methods (Figure A1).

**FIGURE 3:**
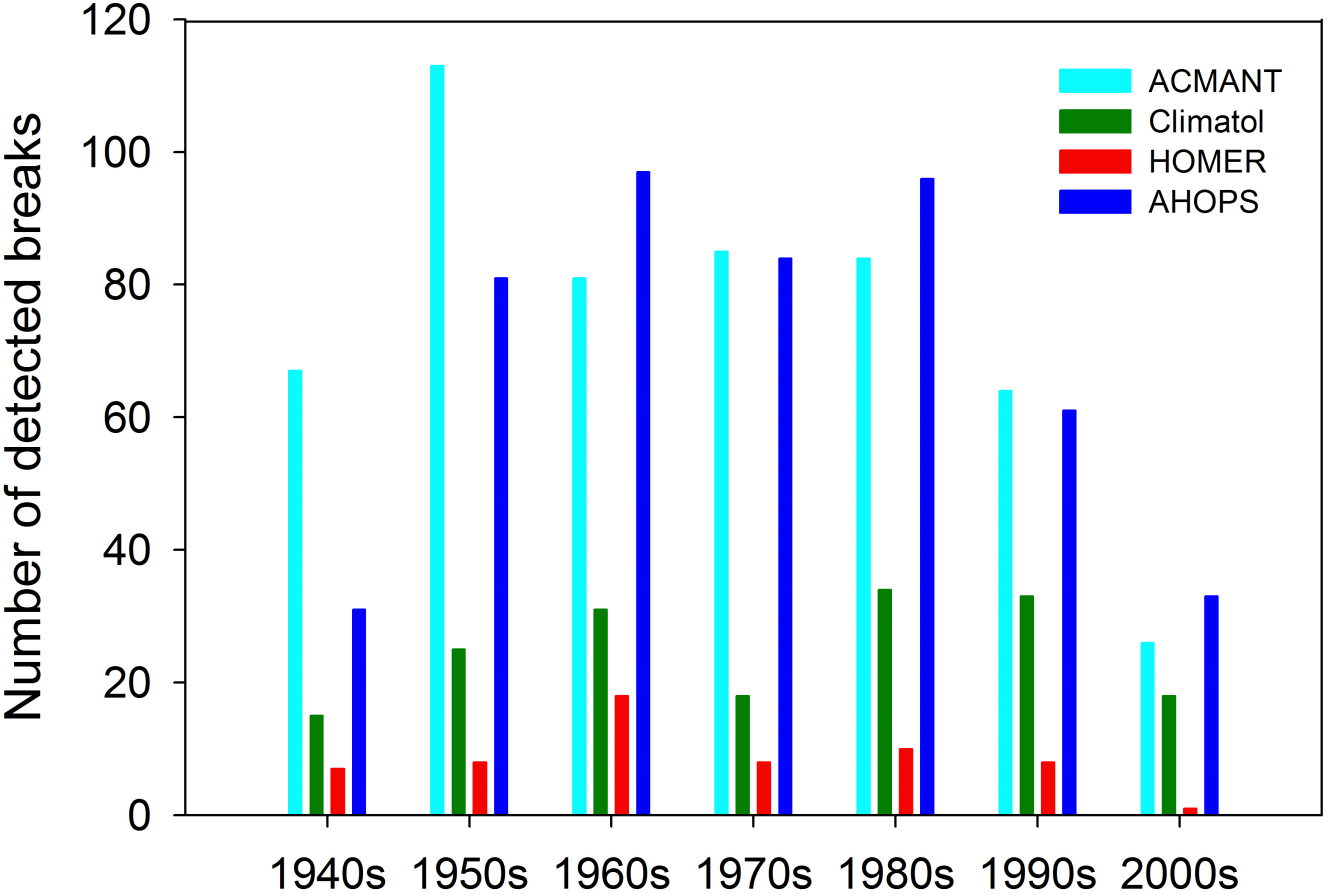
Break detection count summary for the four methods for sub‐IENet by decades (the 1940s start in 1941)

**FIGURE A2:**
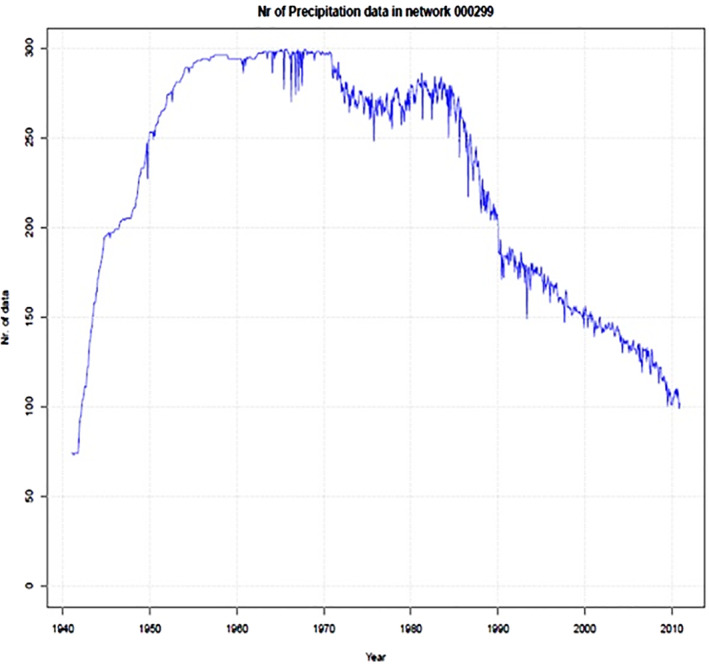
Summary of the overall intact and absent monthly data for the 1941–2010 period of the 299 series of the IENet precipitation dataset. The blue line denotes the greater number of available records for the 1950s–1980s relative to the earlier and later decades

Figure 3 shows the temporal evolution of the detected break frequency for each of the four methods across our base period. It can be seen that fewer breaks were detected in the first decade and the last two decades of the study period, which largely coincides with the temporal evolution of station density (Figure A2). The temporal distribution of break frequency differs considerably according to homogenization methods, for example, the highest number of breaks were detected for the 1960s with HOMER, for the 1950s with ACMANT, for the 1960s and 1980s with AHOPS and for the 1980s with CLIMATOL. Nevertheless, the overall temporal variation of break frequency between 1950 and 1990 is small, while the mean break frequency varies greatly according to homogenization methods. While the total number of detected breaks is only 60 with HOMER, it is approximately 8 times as many with ACMANT and AHOPS (499 and 483, respectively). CLIMATOL detected much more breaks (172) than HOMER, but much less than ACMANT and AHOPS.

In spite of the big differences in the frequency of detected breaks, several concordant breaks were detected by all or by most of the methods. There are 116 occurrences in sub‐IENet when a break was detected with at least three methods within a 24‐month period. Note that in the examination of detection coincidences metadata was considered as an additional break detection method for station series with available metadata. Table 1 presents the list of these events, showing the dates of the detected breaks with all the homogenization methods and the related metadata we found associated with the station history. Station history was available for 98 entries of Table 1, and in 69 cases (70%) of them the statistically detected breaks are connected to coincidental metadata.

**TABLE 1:** List of breaks detected concordantly by year and month for at least three homogenization methods of HOMER, ACMANT, AHOPS, Climatol and metadata for sub‐IENet; n for year and month indicates no detection

Method	Homer		ACMANT		AHOPS		Climatol		Metadata explanation
Station ID	Year	Month	Year	Month	Year	Month	Year	Month	
ID1004	1954	12	1956	9	1954	12	1955	10	Dec 1955: Switch to new station Dec 1955
ID1018	1949	12	1949	11	1949	12	n	n	No station history available
ID1034	n	n	1956	5	1956	12	1957	2	Sep 1956: New station established at new location
ID1036	n	n	1952	10	1952	12	n	n	Mar 1952: Funnel pipe broken
ID1042	2000	12	2002	12	2002	12	n	n	n
ID108	n	n	1988	8	1989	12	n	n	May 1987: Gauge replaced
ID109	1956	9	1957	1	1956	12	1957	1	May 1956: New observer
ID109	1960	12	1960	12	1961	12	1962	4	n
ID1137	1948	1	1948	4	1948	12	1948	4	Mar 1949: Station moved
ID1137	n	n	1957	2	1958	12	1957	2	Feb 1957: Funnel broken
ID1210	n	n	1980	7	1980	12	1980	7	No station history available
ID1240	n	n	1996	5	1996	12	1996	3	Sep 1996: Too sheltered note
ID1304	n	n	N	n	1961	12	1961	12	Jul 1962: Tree encroachment recorded
ID1310	n	n	1981	4	1982	12	1981	4	No station history available
ID1431	1962	12	1963	1	1962	12	1963	10	Jan 1963: Rain measure broken. Young tree which had grown within 10 ft of gauge had to be removed
ID1431	1967	12	1967	8	1967	12	1968	3	n
ID1437	n	n	1959	7	1958	12	1959	8	No station history available
ID1437	n	n	1965	6	1965	12	1966	5	No station history available
ID1514	1946	3	1948	3	1947	12	1948	3	Apr 1946: Gauge levelled
ID1519	n	n	1986	2	1985	12	1986	10	n
ID1529	1965	12	1966	12	1963	12	1967	8	Sep 1965: New mm measure issued, previously measured in inches
ID1529	1968	12	1968	11	1968	12	1968	11	Feb 1969: Gauge replaced
ID1604	n	n	1984	9	n	n	1983	9	Jul 1983: Gauge replaced
ID1605	1963	12	1963	9	1962	12	1963	9	n
ID1605	1967	10	1967	9	1967	12	1967	9	Nov 1967: Gauge replaced
ID1605	n	n	1983	7	1982	12	n	n	May 1982: New gauge installed 50 m from original site
ID1605	n	n	1997	5	1997	12	1997	5	n
ID1605	n	n	2006	2	2005	12	2006	2	n
ID1612	1962	12	1964	12	n	n	1964	11	Nov 1964: Rim of gauge out of shape and incorrect height above ground
ID1619	1946	12	1947	8	1946	12	1947	6	n
ID1619	n	n	1958	1	1957	12	n	n	Mar 1958: 5″ measure half inch capacity issued
ID1619	n	n	1969	10	1969	12	n	n	Nov 1968: Gauge replaced
ID1637	n	n	1991	4	1990	12	1990	5	n
ID1637	n	n	1996	12	1997	12	1998	3	Mar 1998: Gauge exposure issues noted; gauge replaced and not read very often
ID1712	n	n	1953	12	1953	12	1954	2	n
ID1714	1978	12	1978	9	n	n	1978	9	n
ID1729	n	n	1959	5	n	n	1958	4	Apr 1958: New rain measure issued. 1960 readings low for the past 2 years
ID1828	n	n	1974	9	1974	12	1974	11	n
ID1829	n	n	1944	2	n	n	1944	2	Mar 1944: Leaking gauge replaced
ID1829	n	n	1961	12	1961	12	1961	12	n
ID19023	n	n	2003	6	2003	12	2003	6	n
ID1929	1958	12	1959	5	1959	12	1959	5	From Jan 1954: 6‐year gap in inspection reports
ID1940	n	n	1968	4	1967	12	1967	11	Nov 1965: Daily readings from this date, weekly readings prior to this
ID19723	n	n	2003	1	2001	12	2003	6	No station history available
ID19923	n	n	1998	12	1997	12	1997	12	n
ID2005	n	n	1983	9	n	n	1983	10	Dec 1983: Gauge moved
ID2112	n	n	1959	7	n	n	1961	7	May 1961: New gauge installed, previous gauge had a shallow funnel
ID2127	n	n	1954	12	1954	12	n	n	Sep 1955: Rain measure reported broken and replaced
ID2223	1966	3	1966	4	1965	12	1966	4	Apr 1966: Defective gauge; change from 8″ to 5″
ID2227	n	n	1994	11	1992	12	n	n	Between 1984–1998: Intermittent gauge and readings issues. New observer 1998
ID2229	n	n	1963	1	1962	12	n	n	Aug 1963: Outer can leaking report. Jul 1964: Gauge replaced
ID2231	n	n	1975	7	1975	12	1975	8	No station history available
ID2337	n	n	1955	9	1955	12	1955	1	No station history available
ID2404	n	n	1967	7	1966	12	n	n	Apr 1967: Gauge rim raised
ID2406	n	n	1975	12	1975	12	1975	9	n
ID2427	n	n	1955	6	1955	12	n	n	Sep 1955: Inspection report, suspected errors in data since 1948
ID2514	1949	12	1949	12	1950	12	1950	2	Jan 1950: Gauge replaced
ID2635	1976	6	1975	5	1975	12	1976	5	Jul 1976: Gauge dug up and re‐levelled
ID2635	1997	12	1998	5	1997	12	1998	5	n
ID2731	n	n	1958	4	1957	12	n	n	n
ID2737	n	n	1984	5	1983	12	1984	1	Mar 1984: Shaded by trees note. Gauge moved slightly in 1987
ID2802	n	n	1997	2	1997	12	1996	9	Jun 1996: Gauge moved
ID2802	n	n	2005	2	2004	12	2005	2	1998–2010 lots of accumulations
ID3035	1986	12	1987	2	1986	12	1985	9	No station history available
ID3127	1967	12	1967	12	1967	12	n	n	From 1964: 5‐year gap in inspections
ID3135	1983	9	1984	5	1983	12	n	n	Aug 1984: Gauge replaced
ID3223	n	n	1947	3	1946	12	n	n	May 1946: Fencing repairs report
ID3223	n	n	1951	2	1951	12	n	n	n
ID3513	1972	12	1973	8	1972	12	n	n	Site moved 1971 as shaded by trees
ID3519	1982	12	1983	6	1983	12	1983	6	n
ID3604	1990	7	1991	11	1989	12	1991	11	n
ID3604	1994	9	1994	11	1994	12	1994	4	Oct 1994: Gauge replaced and tree shelter noted
ID3704	n	n	1969	2	1969	12	1969	6	Jan 1970: 2 rain measures received report
ID4129	n	n	1979	10	1979	12	1979	10	Aug 1979: Gauge moved
ID418	n	n	1988	5	1988	12	n	n	Aug 1987: Bad site and gauge reports
ID4223	n	n	1998	1	1997	12	n	n	Aug 1997: Change of exposure report, gauge moved
ID430	n	n	1968	7	1968	12	n	n	Sep 1967: Gauge slightly off level report
ID4819	n	n	1969	8	1968	12	1969	8	Jul 2001: Gauge moved slightly, moved again 1/4 mile in 2002
ID4819	n	n	2001	1	2001	12	2001	1	Jul 2000: Gauge moved
ID5012	n	n	1964	8	1965	12	n	n	Feb 1964: Gauge and recorder moved
ID5012	1997	12	1998	12	1997	12	1998	4	n
ID541	n	n	1994	3	1993	12	1994	3	No station history available
ID542	1960	12	N	n	1962	12	1960	12	Aug 1960: Damaged gauge replaced
ID542	n	n	1974	8	1974	12	n	n	Sep 1974: Leaking rain gauge replaced
ID617	1949	12	1949	3	1948	12	n	n	No station history available
ID626	1975	8	1977	4	1975	12	1976	11	Sep 1975: New site (100 yard move)
ID626	1980	7	1980	8	1980	12	1980	8	Aug 1980: Gauge replaced and new observer
ID636	n	n	1958	4	1957	12	1958	4	No station history available
ID703	n	n	1956	3	1956	12	1956	12	Jun 1956: Leaking gauge replaced. No mention of daily gauge, it was monthly mountain gauge that was leaking
ID705	n	n	1978	2	1978	12	1977	9	Mar 1978: Conifer shaded gauge moved
ID7060	n	n	1957	2	1956	12	1956	11	n
ID834	1943	12	N	n	1945	12	1943	11	Oct 1944: Gauge leaking report
ID834	1951	12	1952	3	1951	12	1952	3	Mar 1952: Leaking gauge report, gauge leaking 1944–1952
ID836	n	n	1948	1	1948	12	n	n	Mar 1948: Gauge replaced
ID836	n	n	1977	2	1976	12	1977	2	n
ID844	1970	4	1970	10	1970	12	n	n	May 1970: Guard rail broken and too near gauge. Both observers ill
ID844	1979	12	1980	5	1979	12	n	n	n
ID844	n	n	1998	5	1997	12	1998	5	Jun 1998: Gauge replaced
ID9005	n	n	2003	10	2001	12	2003	10	n
ID9103	n	n	1956	3	1955	12	n	n	Nov 1955–Mar 1956: Problems reported with readings. New observer 1956
ID9103	n	n	1969	3	1968	12	n	n	Dec 1968: Low readings report for the month of December
ID9120	n	n	1989	12	1990	12	1990	11	No station history available
ID922	1966	12	1966	3	1965	12	n	n	No station history available
ID9303	n	n	1976	7	1976	12	1976	7	n
ID9303	n	n	1982	11	1981	12	n	n	Mar 1983: No. 3 Reading low for a number of years Suspect leaking Gauge replaced
ID9403	n	n	1991	4	1990	12	1991	5	n
ID9403	1994	12	1995	6	1995	12	1995	4	Sep 1995: Slight deterioration of station noted
ID9503	1956	5	1954	10	n	n	1954	9	Sep 1954: Number of issues with gauges reported. No 5 gauge replaced 1956
ID9503	1957	12	1957	12	1957	12	1957	10	Jan 1957: Rain measure broken and replaced report
ID9503	n	n	1991	4	1990	12	1991	1	n
ID9505	n	n	1955	8	1955	12	1955	10	No station history available
ID9604	n	n	1993	4	n	n	1993	9	Jul 1993: Gauge replaced
ID9605	n	n	1955	9	1955	12	1955	10	No station history available
ID9705	n	n	1982	6	1981	12	1982	6	n
ID9904	1956	5	1956	1	1955	12	n	n	1956 gauge readings too low, definitely interfered with
ID9940	n	n	1980	12	1980	12	1982	3	Jul 1980: Stolen funnel replaced. Jul 1980: New site
ID99950	n	n	1983	3	1982	12	1983	5	No station history available (Met Office, United Kingdom NI station)
ID99990	1960	12	1960	12	1960	12	1960	12	No station history available (Met Office, United Kingdom NI station)

*Note:* Metadata is considered as an additional break detection method; n in metadata explanation denotes no metadata support.

#### 3.1.2 Break magnitudes

**FIGURE 4:**
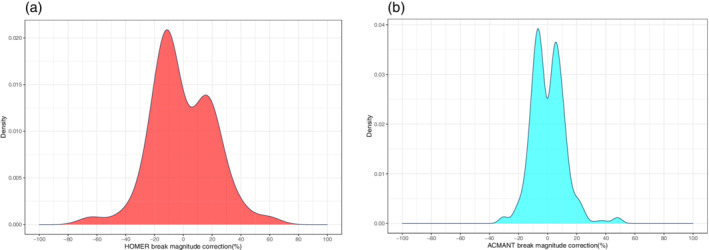
Kernel density plots illustrating the range and distribution of break magnitude corrections by (a) HOMER and (b) ACMANT. The difference in y‐axes scales reflects the different detection frequencies between the two methods

Break magnitudes indicating the size of adjustment terms are monitored in the homogenization with HOMER and ACMANT. Most of the adjustment terms applied by HOMER approximate a normal distribution, although there are some outlying values (Figure 4a). This is also largely true for the ACMANT adjustment terms (Figure 4b). In both parts of Figure 4 the normal distribution is truncated around zero, due to the missed breaks of insignificant magnitudes (see also Menne *et al*., 2009; Lindau and Venema, 2019).

With HOMER, both the largest positive and negative shifts were found for Glenvickee (Caragh River Area) in 1963 (with ratio 1.59) and 1967 (with ratio 0.67), respectively. This inhomogeneity of Glenvickee precipitation between 1963 and 1967 was also detected with ACMANT, indicating 46–47% positive, non‐climatic bias of the raw data for that period.

**FIGURE 5:**
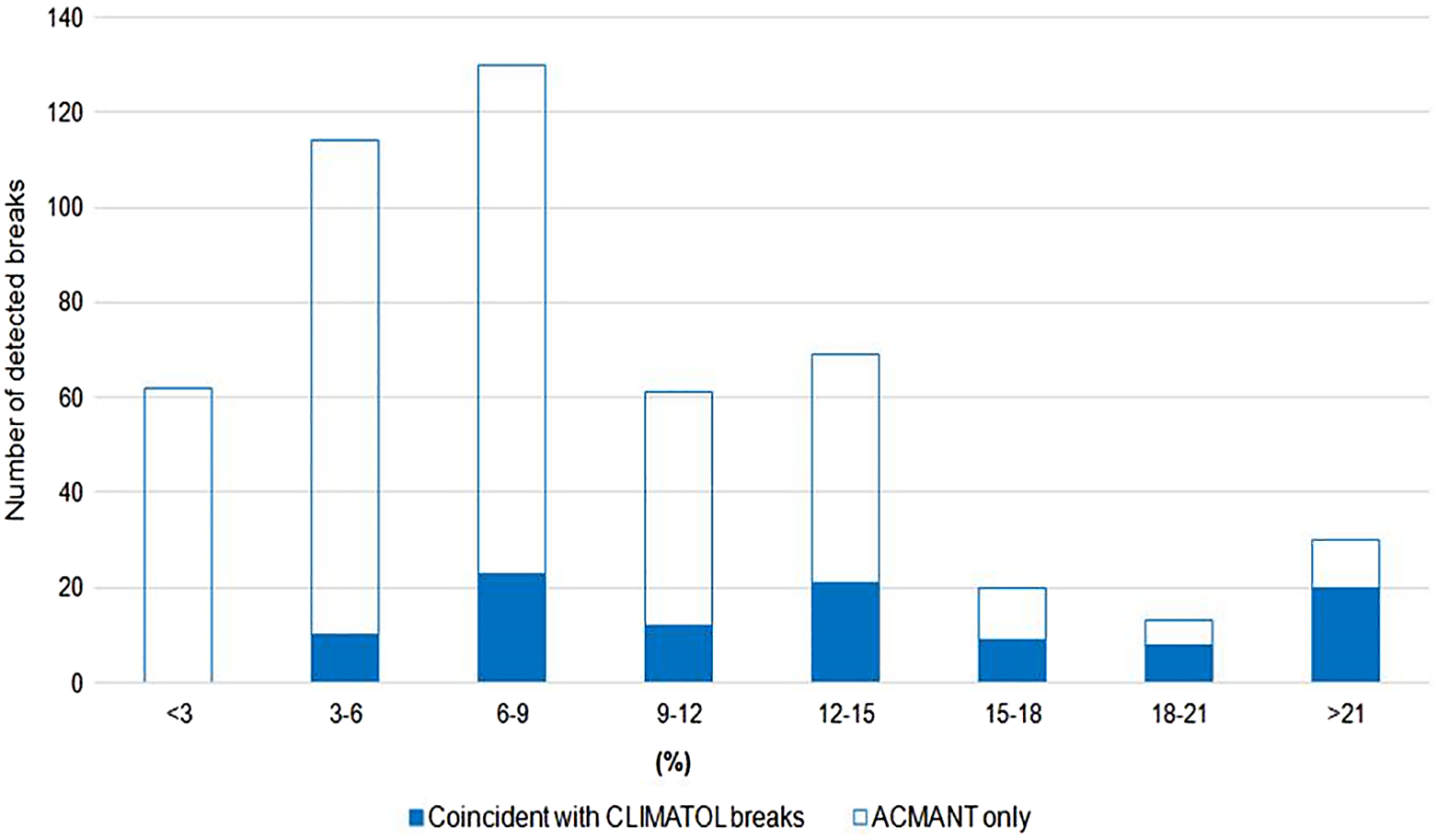
Break size distribution for ACMANT detected breaks. Blue bars indicate coincidences with CLIMATOL breaks, white bars those for ACMANT only

Figure 5 shows the magnitude distribution for all breaks detected with ACMANT, and for breaks detected with both ACMANT and CLIMATOL. The largest frequency was found for breaks of 6–9% change, likely because the detection of smaller breaks is more difficult for lower SNR. The proportion of breaks detected with both ACMANT and CLIMATOL monotonously increases with break magnitude when the amount of these breaks is compared to that of all ACMANT detected breaks.

### 3.2 Homogenization with the use of the whole IENet (910 stations)

The operation of ACMANT and CLIMATOL remained stable in homogenizing the datasets with large ratios of missing data. The results are reliable in the sense that visible large homogenization errors have not appeared.

Overall break‐frequency has increased with the use of the denser dataset compared with the results in section 3.1, in the case of ACMANT (CLIMATOL) with 6% (13%). The number of homogeneous series decreased very slightly with CLIMATOL (from 178 to 172), while it increased with ACMANT from 65 to 76. In the case of some series where breaks were detected using sub‐IENet, inhomogeneities are no longer indicated with the use of the denser whole IENet. This latter change was noted for 20 (34) series with CLIMATOL (with ACMANT).

The number of series with multiple breaks is slightly increased both with CLIMATOL and ACMANT. Also, the break positions often changed markedly by comparison with sub‐IENet, particularly with ACMANT. The change in the number of detected breaks for individual time series is recorded for 63 series (21%) with CLIMATOL and in 177 series (59%) with ACMANT.

The stability of break positions for large breaks and concordant break detections was examined. Table 1 includes 83 breaks detected concordantly with ACMANT, CLIMATOL and at least one more method with the use of sub‐IENet. Of these breaks, 72 (87%) were detected with CLIMATOL in the same or nearby position with the use of the whole IENet, and in this case the ACMANT results are similar (71 breaks, 86%). “Nearby” here means that the coincidence with the metadata and at least one more statistically detected break remained in the same 24‐month period defined for the entries of Table 1. By way of further comparison, of the 73 breaks recording a magnitude of at least 15% according to ACMANT detection with sub‐IENet, when whole IENet was used 57 (78%) of these were detected at the same or nearby positions as for the smaller network.

**FIGURE 6:**
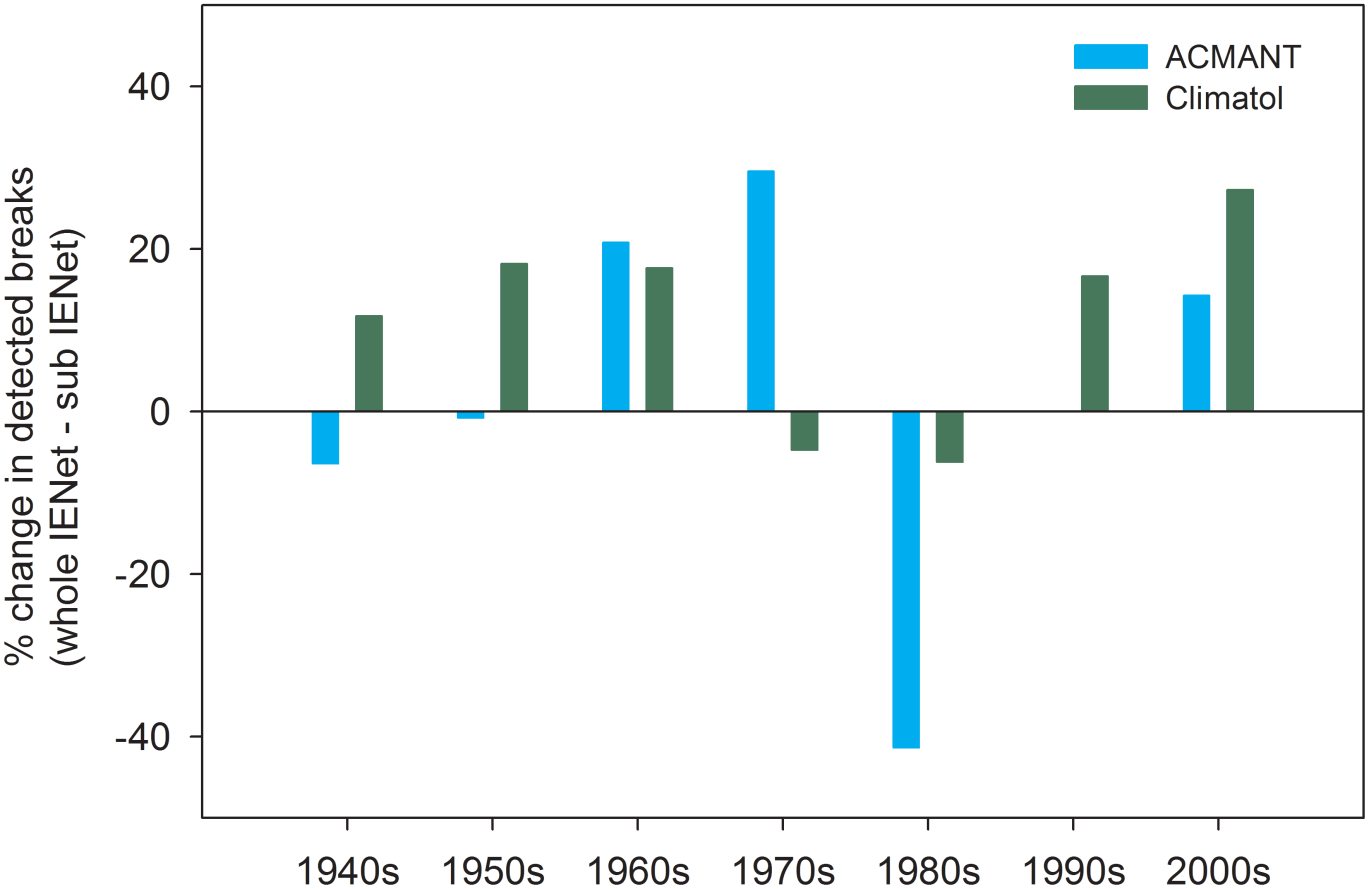
Break detection frequency change (percentage) by decades, for whole‐IENet by comparison with sub‐IENet (the 1940s start in 1941)

**FIGURE 7:**
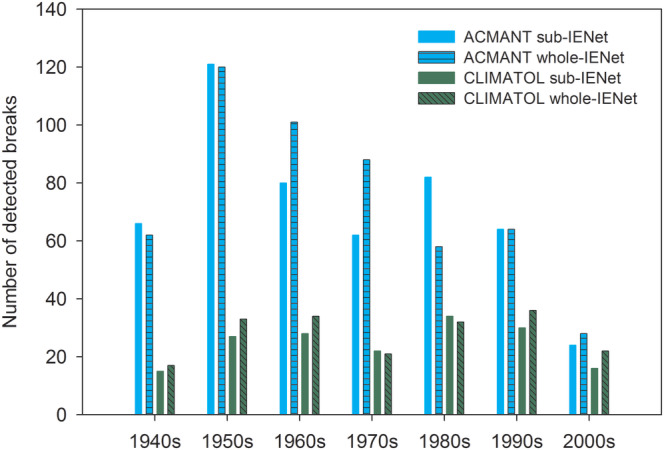
Histogram break detection count summary for the two methods (ACMANT and CLIMATOL) for the sub‐IENet and whole‐IENet by decades

**FIGURE A3:**
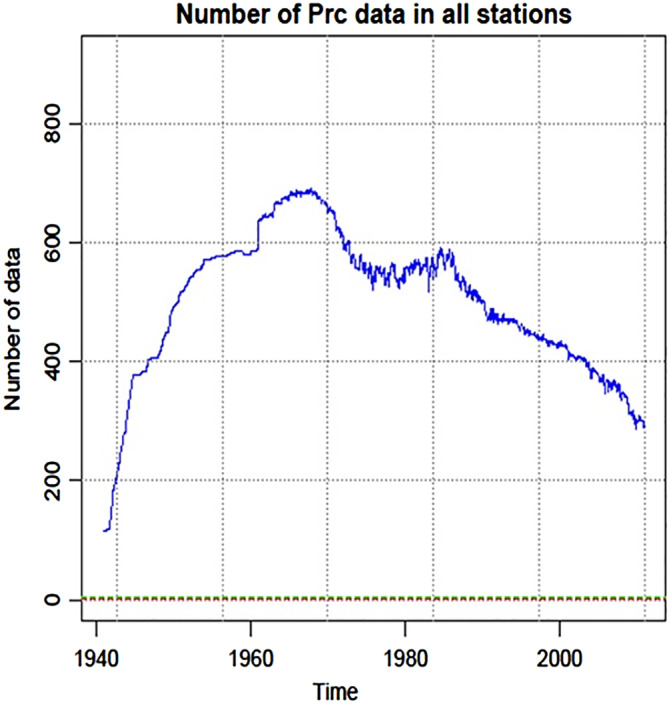
Summary of the overall intact and absent monthly data for the 1941–2010 period of the 910 series of the whole‐IENet precipitation dataset. The blue line denotes the greater number of available records for the 1950s–1980s relative to the earlier and later decades

The temporal distribution of detected breaks changed with both CLIMATOL and ACMANT in comparison with the sub‐IENet results, but with notably different patterns (Figures 6 and 7). CLIMATOL detected about 20% more breaks in the first three decades and last two decades of the period, while break frequency remained practically unchanged between 1970 and 1990. By contrast, with ACMANT the break frequency increased in the 1960s and 1970s, decreased in the 1980s, and hardly changed in the other sections of the period. As with sub‐IENet, the temporal evolution of break frequency is partly coincident with that of station density and hence with the overall data availability in whole‐IENet (Figure A3).

## 4 DISCUSSION

We have found large differences in the ratio of homogeneous series according to the homogenization methods applied. With HOMER, the majority of the precipitation records seem homogeneous, while in ACMANT and AHOPS the majority seem inhomogeneous. By comparison CLIMATOL returned a more intermediate assessment. Note that neither the ratio of homogeneous series, nor the number of detected breaks indicates the accuracy of homogenization, which is characterized by the residual deviation from the true climate signal after homogenization. However, the detection statistics give useful indications in relation to the strengths and weaknesses of homogenization methods used. One likely explanation of the divergence in the estimated ratio of homogeneous series is that in many time series the inhomogeneities are small or relatively small; hence, the SNR is too low for accurate break detection. Breaks of low SNR mean that the dataset cannot be separated into the two classical clusters of a fully homogeneous subset and a subset with significant inhomogeneities, but rather indicate that there is an intermediate cluster in which not all of the homogenization methods find breaks. The results show that the frequency of detected breaks with ACMANT and AHOPS were approximately 8 times as high as than with HOMER. A larger number of detected breaks with ACMANT than with HOMER has also been reported in a comparative study on the homogenization of temperature data in the Spanish Pyrenees (Pérez‐Zanón *et al*., 2015). Therefore, it would appear that this kind of difference is an artefact of the homogenization methods themselves, rather than the characteristics of the data subjected to homogenization. Another outcome supporting this finding is that in a study homogenizing precipitation data in Norway, MASH was found to detect six times as many breaks than HOMER (Lundstad *et al*., 2017). It is also worth noting that a relatively high false alarm rate has already been reported in relation to the use of both ACMANT and MASH (Killick, 2016), that is, that in testing the accuracy of daily temperature homogenization methods, these methods more often than not introduced unnecessary adjustments to homogeneous series compared with other methods.

The relatively high frequency of detected breaks in the IENet dataset in comparison with other precipitation datasets (Domonkos, 2015 and references therein) likely stems from the relatively high spatial correlations of the IENet dataset which provide high SNR for the relative time series examined. However, the SNR is not necessarily high for all the inhomogeneities, or at least the frequent change in the detection results associated with the changes of the reference networks (i.e., sub‐IENet or whole IENet) suggests this. With the use of the denser whole IENet, the number of breaks for individual time series changed in comparison with the sub‐IENet results for 23% (59%) of the series using CLIMATOL (ACMANT). ACMANT detected more breaks and more small breaks than CLIMATOL for instance, and it may offer one explanation of the particularly high instability of the ACMANT detection results. It is known that for low SNR breaks the recognition of the breaks and the estimation of break positions are characterized by high uncertainty (Menne and Williams, 2009). This uncertainty has recently been quantified by Lindau and Venema (2016; 2018b). The uncertainty of break detection results for low SNR breaks follows directly from the Caussinus–Lyazrhi Equation (2).

(2) $$Z\left(K\right)=\ln\left\{1-\frac{\sum_{k=1}^{K+1}l_{k}{\left({\bar{T}}_{k}-\bar{T}\right)}^{2}}{\sum_{i=1}^{n}{\left(t_{i}-\bar{T}\right)}^{2}}\right\}+\frac{2K}{n-1}\ln\left(n\right).$$

In Equation (2) *Z* stands for the Caussinus–Lyazrhi score, *l* denotes the length of homogeneous section *k*, and upper stroke denotes the arithmetical average. In the break detection process the *K* with the lowest *Z* is always selected. The numerator within the first logarithmic expression is defined as external variance. This external variance always increases with *K* such that *Z* would decrease monotonously without the second term, the so‐called penalty term. The core idea of the Caussinus–Lyazrhi criterion is that if a large break splits the series into two sections then the external variance increases faster than the change of the penalty term, while for insignificant breaks and noise the increase of external variance is relatively small. As a consequence, most of the insignificant breaks do not appear as detected breaks and it limits *K*. When small breaks or unusually shaped noise structures are detected as breaks, they may result in a similar increase of the external variance to that of the penalty term. It cannot be excluded either theoretically or for practical homogenization tasks that the *Z* score for *K* = 0 is similar to a *Z* of *K* > 0, and then a little change in the reference series selection can change the detection result. Similarly, the *Z* score can be similar for two different *K* > 0 values. Lindau and Venema (2018b) analyse this problem from other perspectives and based on this recommend an alternative method for estimating *K*; however, their suggested method has not yet been used in practical time series homogenization.

The results for IENet break detection show that the break positions for large breaks are mostly stable as predicted by Equation (2). Note that the SNR also depends on factors other than break size. These factors are the shape and magnitude of background noise and the persistence of the bias resulting from the break. The role of these factors may explain that a certain ratio of the breaks with at least 15% change still showed instability.

With the increase of station density an increase of the frequency of detected breaks is expected. Detection of smaller breaks is favoured due to the higher spatial correlation and SNR. Our results show only a very slight increase in the break frequency when the use of sub‐IENet was changed to the use of the whole IENet. One unexpected aspect of the results is that the ratio of series qualified to be homogeneous by ACMANT increased with the use of the denser dataset from 21.7 to 25.4%. Although this increase is small, the sign of change is opposite to what might be expected, hence it is a likely indication that in some cases spatial climatic differences are erroneously considered to be inhomogeneities when the sub‐IENet was used in ACMANT homogenization. The base model of relative homogenization presumes that the climate signal is identical for stations where the series are homogenized together (see section 2.3). Persistent spatial differences in weather and climate are often considered in break detection methods via the calculation of first order autocorrelation from the observed data (Wang, 2008; Li and Lund, 2012). A weakness of this approach is that the empirical autocorrelation of *T* series often considerably differs from the autocorrelation of the weather term *ε* (Equation (1)). However, the empirical parameterization of ACMANT also has a weakness, namely the representativeness of the parameterization for climatic regions and climatic variables not tested is unknown.

In explanatory research of station history it is important to provide reliable break detection results. Our results show that ACMANT and AHOPS are not the best choices for this purpose, while HOMER has the particular advantage that potential breaks can be examined interactively during the detection process. Note, however, that there are 15 entries in Table 1 related to metadata, which all the homogenization methods detected with the exception of HOMER. This suggests that HOMER might miss the detection of some significant breaks during precipitation homogenization. It has been suggested that it might be easier for HOMER to find breaks in the middle of time series rather than at the beginning or end for temperature data (Osadchyi *et al*., 2018). However, and in our experience with precipitation data, series missing contiguous data blocks early or late in a series pose significant challenges for HOMER. For this reason the WMO Task Team on homogenization recommends a missing data tolerance of 15 years for HOMER (WMO, 2017), while, for example, Météo‐France works with a 10–15% missing value tolerance depending on the length of series in operational homogenization using HOMER (B. Dubuisson Météo‐France, September 2016, personal communication). A further problem found with HOMER is that its inbuilt calculation of spatial correlation sometimes fails when data gaps are present in time series, thus the use of the geographical distance option is recommended for the HOMER homogenization of incomplete series. All these, together with another problem with HOMER discussed by Gubler *et al*. (2017) and Domonkos (2017) indicate that HOMER should be revised and tested.

Metadata use may elevate the efficiency of homogenization, particularly for areas and periods of spatially rare observations. The quantification of this benefit has not been solved yet by the scientific community. Some basic problems of a possible quantification for metadata are the varied completeness of metadata according to networks and stations, and also the varied individual value of the pieces of metadata (e.g., in Table 1; “Switch to new station” at ID1004 is likely more important than “Gauge replaced” at ID108).

In spite of the frequent instability of break detection results with ACMANT, this method gives the most accurate homogenization in terms of residual root mean squared error and trend bias (Venema *et al*., 2012; Killick, 2016; Guijarro *et al*., 2017), at least when the efficiency of homogenization methods is tested without metadata use. An explanation for this seeming contradiction is that in the case of small breaks the homogenization results are generally imperfect with any *K* when the Caussinus–Lyazrhi criterion are applied, hence the impact of the number of detected breaks on the accuracy of homogenization is not decisive. However, this and many other aspects of the results suggest that more tests with realistic test datasets could only consolidate our knowledge about the reliability and potential accuracy of homogenization products.

## 5 CONCLUSIONS

In this study the break detection results with HOMER, ACMANT, AHOPS and CLIMATOL for a subset of 299 series (sub‐IENet) were compared and analysed partly with the help of the series from a larger network of 910 series from Ireland and Northern Ireland (whole‐IENet). The characteristics of the monthly total precipitation data of whole‐IENet, that is, a fairly dense network allied to the climatic characteristics of a maritime region with low amplitudes of variation between series result in relatively close geographic proximities and high correlation coefficients among many series. Although comprising fewer stations, sub‐IENet is still a dense subset with high spatial correlations. These properties of the data are excellent for the application of relative homogenization methods. Our main findings are as follows.

We have found 116 concordant breaks indicated with at least three homogenization methods including metadata analysis. This together with some other aspects of the results suggests that the break frequency for whole and sub‐IENet is higher than in several other European precipitation datasets. However, spatial correlations are generally higher in whole and sub‐IENet than in many other precipitation datasets. For time series highly correlated with other series of nearby stations, smaller breaks can be more readily detected than is the case for less highly correlated series; and hence the small breaks detected might give the unfounded impression that the time series of whole and sub‐IENet are less homogeneous than those of other precipitation datasets. Additionally, the ratio of series found to be homogeneous and the frequency of detected breaks are strongly method dependent, thus a valid comparison between detection statistics of different station networks would need the use of the same break detection methods.

The homogenization of whole and sub‐IENet proved that ACMANT and CLIMATOL can easily be applied for the homogenization of large datasets. With these two methods, varied lengths of time series or data gaps within time series do not affect neither the operation of the software programmes nor the reliability of the homogenization results. The operation of HOMER is characterized by limited missing data tolerance, while AHOPS does not detect breakpoints when there are not enough observations to build a reference series, though the correction can handle gaps in the time series.

The differences between the frequencies of detected breaks according to homogenization methods are much larger than the differences between the accuracies of the climate signal reconstruction (Guijarro *et al*., 2017), due to the different strategies of homogenization methods in using detected breaks within the homogenization procedure. The comparison of our results with some other studies indicates that the instability of break detection results does not have direct relation to the accuracy of homogenized time series.

The break frequency results of CLIMATOL seem to be the closest to the reality among the four methods examined here; therefore, we recommend the use of CLIMATOL break detection results when the aims of the homogenization include the analysis of station histories.

## Data Availability

Regular updates on the software used and where to obtain it can be found, for example, on the website of the Task Team on Homogenization (OPACE2, WMO Commission for Climatology; URL: http://www.climatol.eu/tt-hom/). A version of the metadata used (with observer personal information removed) can presently be obtained on request from Met Éireann (Mary Curley). At the time of publication there is an aspiration to host this on the Met Éireann website in the near future.
